# Effectiveness of Post-Hospital Intensive Residential Rehabilitation after Acquired Brain Injury: Outcomes of 256 Program Completers Compared to Participants in a Residential Supported Living Program

**DOI:** 10.1089/neu.2018.5944

**Published:** 2019-12-11

**Authors:** April R. Groff, James Malec, Debra Braunling-McMorrow

**Affiliations:** ^1^ Learning Services Corporation, Lawrenceville, Georgia.; ^2^ Rehabilitation Hospital of Indiana, Indianapolis, Indiana.

**Keywords:** brain injury, outcomes, rehabilitation

## Abstract

Post-hospital residential brain injury rehabilitation outcomes research is a complicated undertaking because of the custom-tailoring of interventions needed to meet the complex and unique need of each individual. As such, there tends to be great variability across program settings, which generally limits large-scale intervention studies. Growing literature demonstrates that post-hospital residential programs are beneficial. The main criticisms of this work include the absence of randomized-controlled studies, lack of clear definition of treatment types/settings, and small sample sizes. This study is a retrospective analysis of program evaluation data for a large, multi-site, national provider of post-hospital residential brain injury rehabilitation services. Specifically, outcome of participants completing Intensive Residential Rehabilitation (IRR) were compared to participants in the Residential Supported Living (RSL) program. Results demonstrate that participants in the IRR program improve and that participants in the RSL group preserve functional ability over time, suggesting that each program is effective in achieving its intended outcome. The IRR treatment group achieved significantly better outcomes than those in the same setting not receiving the intervention. To isolate treatment effects of IRR, a subsample of participants across program types were matched on time post-injury, age, and sex. The treatment effect of IRR was strengthened in this analysis, suggesting that chronicity alone does not account for the variance between the two groups.

## Introduction

Brain injury is a complex and consequential public health issue with significant clinical and cost implications. An estimated 9 million Americans currently live with a brain-injury–related disability.^1^ Each year, there are more than 3.3 million individuals in the United States diagnosed with an acquired brain injury,^2,3^ with an estimated annual cost to society in excess of $100 billion.^2,4^

Given that hospital stays in recent years have shortened, individuals with moderate-to-severe brain injuries are being discharged needing more assistance, care, and intervention because of more significant medical, cognitive, physical, and behavioral problems at discharge than observed in earlier decades. This has resulted in a heavy demand for an effective continuum of post-hospital services for individuals with these injuries. The burden of care is often too excessive for successful management in the home or community setting and has increased the demand for developing effective post-hospital residential rehabilitation programs to maximize functional outcomes for these individuals. Simultaneously, coverage and reimbursement for needed services after hospital discharge is becoming increasingly sparse, yielding a gap in our healthcare that ultimately leads to both increased disability and additional financial impact on society.

Demonstration of effective rehabilitation after brain injury is paramount for restoring function and productivity of the individuals impacted by an individualized array of chronic physical, cognitive, psychological, behavioral, and functional impairments. It is also important to justify the necessity of treatment and reimbursement for such treatment from third-party payers.

Outcomes research in post-hospital brain injury rehabilitation is complex and often a complicated undertaking for a variety of reasons. The nature of brain injury rehabilitation requires the custom-tailoring of interventions to meet the complex and unique need of each individual. As such, there tends to be great variability across program settings, which generally limits large-scale intervention studies containing a viable control group.

The current body of literature on effectiveness of post-hospital brain injury rehabilitation programs has established an optimistic base for continued study. Several review articles examining the effectiveness of the post-hospital rehabilitation programs have been completed to date.^5–7^ The combined results of these reviews establish evidence that post-hospital brain injury services are beneficial to the individuals served. Significant pre-post gains in functional ability have been demonstrated across program setting and type.^5,8–14^ Past studies have also demonstrated that individuals who received post-hospital rehabilitation services within the first year of injury saw the greatest gains,^15–18^ and that even those who are exposed to post-hospital rehabilitation services when they are a year or more post-injury still experience significant functional improvement as a result of participation.^9,16–22^ This is likely attributed to the fact that in the post-hospital phase, treatments target both the recovery of lost function/ability (generally thought to be achievable during the initial period of spontaneous recovery) and the teaching of compensatory techniques and skills that can improve function despite residual deficit. Whereas those receiving treatment earlier may benefit from both aspects of treatment, those receiving treatment after the first year may benefit more from the compensatory training components of rehabilitation.

The main criticisms of this body of work include: 1) the overall absence of randomized-controlled studies (RCTs), which have unwittingly become the perceived gold standard for establishing reimbursement guidelines for healthcare, 2) lack of clear definition of treatment, and 3) small sample sizes.

The present study is a retrospective analysis of program evaluation data for a large, multi-site, national provider of post-hospital brain injury rehabilitation services. Specifically, outcome of participants completing Intensive Residential Rehabilitation (IRR) were compared to participants in the Residential Supported Living (RSL) program.

Programs are defined as follows.

### Intensive Residential Rehabilitation

IRR is an outcome-oriented, goal-directed residential program designed to maximize functional outcomes for adults with acquired brain injury. Intensive skilled individual therapies are provided between 10 and 20 h per week with the goals of maximizing physical ability, cognitive ability, psychosocial/behavioral adjustment, independent living skills, and family/community reintegration, in addition to daily group interventions. The focus of the program is functional improvement over time and generalizing skills from the therapy office to application in real-life community settings.

### Residential Supported Living

RSL is an activity-based residential program designed to preserve current/optimal level of independent functioning by providing customized activity to preserve physical ability, cognitive ability, psychosocial/behavioral adjustment, independent living skills, and family/community reintegration. Focus of the program is on maintaining functional stability over time and improving overall quality of life.

Where many other studies have treatment groups that are comprised of both residential and community-based interventions,^5,8^ this study is focused on the comparison of two residential treatment programs, one focused on intensive rehabilitation and the other focused on maintenance of current status, within the same framework of programs in order to assess the specific effectiveness of IRR. Simultaneously, this study is unique in design that the RSL group is a natural control group, which allows for a comparison between individuals in the same residential treatment programs who are not receiving the IRR intervention to those who are receiving the IRR intervention.

The aim of this study is to 1) examine the characteristics of participants in a national network of post-inpatient brain injury services, 2) compare outcomes of participants across treatment-type groups, and 3) utilize a matched subset comparison of outcomes between treatment groups in order to further isolate treatment effect.

Our hypotheses are centered on the idea that intensive post-acute residential rehabilitation does positively impact outcome for participants, independent of chronicity. Specific hypotheses were as follows: 1) Participation in IRR program is associated with improvement in outcomes, 2) participation in the IRR program results in significant functional improvement relative to participation in RSL program, and 3) the effect of IRR treatment is strengthened when chronicity is more rigorously controlled.

## Methods

A retrospective before/after observational study was conducted. Data were collected from all participants served between 2010 and 2015 within a national network of eight residential and outpatient programs across six states. Upon admission to the programs, participants consented to having their progress and outcome data available for analysis. The Indiana University Institutional Review Board provided exemption for analysis of de-identified data.

The initial sample included 462 cases. Cases with atypical lengths of stay who were in the Intensive Rehabilitation programs were excluded. This included 2 cases with less than 7 days and 35 cases with more than 365 days in the program.

The final sample included 425 individuals in six treatment tracks within the residential rehabilitation setting: 1) Neurorehabilitation (*n* = 161); 2) Neurobehavioral Rehabilitation (*n* = 57); 3) Day Treatment (*n* = 38); 4) Supported Living (*n* = 131); 5) Neurobehavioral Supported Living (*n* = 25); and 6) Day Activity (*n* = 13). These six program types were further grouped into two major program categories: 1) IRR (program types 1–3), in which the overarching goal was to achieve significant gains through an intensive rehabilitation process in the residential setting, and 2) RSL (program types 4–6), in which the goal was to assist the individual in maintaining current status over the long term and enhance quality of life. Characteristics of participants in each program category on admission are described in Table 1.

**Table 1.** Demographic, Injury, and MPAI-4 Variables on Admission by Major Program Category

Variable	Intensive Rehabilitation (*n* = 256)	Supported Living (*n* = 169)
Sex (% male)	89.8	84.6
Mean (SD) age at injury (years)^a^	42.34 (15.16)	34.57 (15.33)
Mean (SD) age at admission (years)	44.38 (14.49)	42.02 (12.69)
Mean (SD) days post-injury at admission^a^	661.11 (1352.11)	2732.07 (3306.12)
Mean (SD) length of stay (days)^a^	114.27 (85.99)	3115.10 (2729.88)
Race/ethnicity		
Caucasian	68.8%	74.0%
African American	7.0%	5.9%
Hispanic	19.5%	14.2%
Asian	.8%	3.6%
Native American	1.6%	1.8%
Other	2.3%	.5%
Injury type^a^		
Closed	84.0%	75.7%
Open	2.0%	7.1%
Stroke	7.4%	4.7%
Anoxia	3.5%	4.1%
Other	3.1%	8.2%
Funding source^a^		
Workers' Compensation	55.9%	56.8%
Commercial/no-fault	13.7%	7.7%
Private pay/lien	16.8%	22.5%
Military	11.6%	4.7%
Public	2.0%	8.3%
Mean (SD) MPAI-4 T score at admission		
Ability Index	51.38 (11.17)	51.32 (11.56)
Adjustment Index	50.67 (12.36)	48.72 (10.93)
Participation Index	48.98 (10.98)	50.47 (10.07)
Total	50.97 (12.02)	50.40 (10.25)

^a^ Significant difference (*p* < 0.05) between groups.

MPAI-4, Mayo-Portland Adaptability Inventory-4; SD, standard deviation.

The primary outcome measure analyzed was the Mayo-Portland Adaptability Inventory-4 (MPAI-4). The MPAI-4^23^ consists of 30-items selected to assess commonly occurring limitations after acquired brain injury. It is divided into three subscales: Ability Index, Adjustment Index, and Participation Index. Lower scores indicate lesser impairment and limitations. Earlier studies have demonstrated satisfactory internal consistency, construct validity,^24–26^ as well as concurrent^27^ and predictive validity^28–30^ for the full measure and its indices. The MPAI-4 has been found to be responsive to the effects of rehabilitation interventions.^28–32^ The MPAI-4 and a manual for its use are freely available on the Center for Outcome Measurement in Brain Injury (COMBI) website (www.tbims.org).

Demographic information, injury-related characteristics, and MPAI-4 consensus ratings were recorded at admission for all 425 participants. MPAI-4 consensus ratings were recorded for the IRR group at discharge (mean length of stay = 113.0 days; standard deviation [SD] = 84.40) to compare pre-treatment and post-treatment scores. MPAI-4 ratings for the RSL group were recorded on admission and annually to assess change over time. For the purpose of this analysis, we compared the admission MPAI ratings to the first annual reassessment ratings for the RSL group.

Differences in demographic, injury-related, and MPAI-4 variables on admission between the two major program categories were examined using *t*-tests or chi square tests, as appropriate. Differences on these variables among the program types in the IRR category were also examined using the same procedures. Differences among program types in the RSL category were not analyzed because of small numbers in two of the program types in this category. Because chronicity (days post-injury) was highly skewed, the log_10_ conversion of this variable was used in all computations. T-scores for the MPAI-4 Total and Index scores were derived with reference to a large sample used to compute T-scores in a previous study^5^ to make results comparable to those obtained in the earlier study. Algorithms for T-score derivation were provided by Inventive Software Solutions. Differences among the two major program categories on MPAI-4 Index and Total scores at discharge were evaluated using analysis of covariance (ANCOVA) with MPAI-4 score on admission, sex, age at injury, and log chronicity as covariates. Using similar procedures, ANCOVA analysis of discharge MPAI-4 scores were also conducted contrasting the program types within the IRR category.

A previous publication also identified the minimal clinically important difference (MCID) and the robust clinically important difference (RCID) for the MPAI-4.^33^ The MCID, defined as the smallest change in score that indicates a clinical significant change in functional status, was determined to be 5 T-score points or 0.5 SD. The RCID, defined as a change in score that indicates a clearly substantial change in functional status, was determined to be 9 T-score points. We compared the proportion achieving an MCID and an RCID between program categories and types.

Time post-injury (chronicity) was much greater for the RSL group than for the IRR group, and this variable was significantly related to outcome. In order to evaluate the degree to which chronicity explained the differences between the two major program categories more rigorously than can be accomplished through covariate analysis, we matched a group of RSL participants to a group of the IRR participants on chronicity. We then compared these two matched groups on the discharge/second assessment MPAI-4 T-scores.

## Results

### Intensive Residential Rehabilitation versus Residential Supported Living comparisons

#### Demographic variables

Group comparisons were made between the IRR and RSL groups across demographic, injury-related, and funding source variables. With regard to demographic variables measured at the time of admission, the IRR and RSL groups differed significantly on age at injury (*t* = 5.12; *p* < 0.001), with those in the IRR group being slightly older at the time of injury. The two groups did not differ significantly on sex, age at admission, or ethnicity. Both groups were predominantly comprised of white male participants. Ninety percent of the IRR participants were male, and 85% of the RSL group was male. The IRR group was 69% Caucasian, and the RSL group was 74% Caucasian.

#### Injury-related variables

With regard to injury-related variables, the IRR and RSL groups were significantly different on log_10_ chronicity (*t* = −11.67; *p* < 0.001), with the RSL group demonstrating higher chronicity (measured as mean days post-injury at time of admission). On average, IRR participants were 1.8 years post-injury compared to 7.5 years for the RSL groups. Both groups were similar in distribution across injury types. Closed traumatic brain injuries were the most prevalent across groups (IRR = 84% and RSL = 76%). Open brain injuries occurred at a higher proportion in the RSL group (7.1%) compared to the IRR group (2%). There were no other significant differences between the two groups on injury-type categories.

#### Funding category

Both groups were similar, in that slightly over half of the participants were injured in work-related accidents and treatment was funded through Workers' Compensation insurance. The IRR group had a significantly higher proportion of participants covered by both other insurance (14%) and military/VA (12%) funding than the RSL group did. The RSL group had a significantly higher proportion of private pay or lien cases (23%) than the IRR group (17%).

#### Baseline measurement

The two groups were compared on mean MPAI-4 T Index and Total scores at admission. There were no significant differences between the IRR and RSL group on mean Ability Index, Adjustment Index, Participation Index, or Total T-scores.

#### Outcome measurement

Total MPAI-4 T-scores at program discharge for the IRR group or second assessment for the RSL group were compared using ANCOVA with MPAI-4 Total T-score on admission, age at injury, and log chronicity as covariates. Program category was significantly related to discharge/second assessment MPAI-4 Total T-score (*F* = 56.97; *p* < 0.001) with a large effect size (partial eta^2^ = 0.12). Admission MPAI-4 Total T-score was highly associated with outcome (*F* = 518.00; *p* < 0.001; partial eta^2^ = 0.57). Other covariates were also significantly associated with outcome: log chronicity (*F* = 33.94; *p* < 0.001; partial eta^2^ = 0.08) and age at injury (*F* = 4.89; *p* = 0.03; partial eta^2^ = 0.01). Effect size for change in the program for the IRR group without reference to the RSL group was very large, that is, around 1 SD (Table 1).

**FIG. 1.**
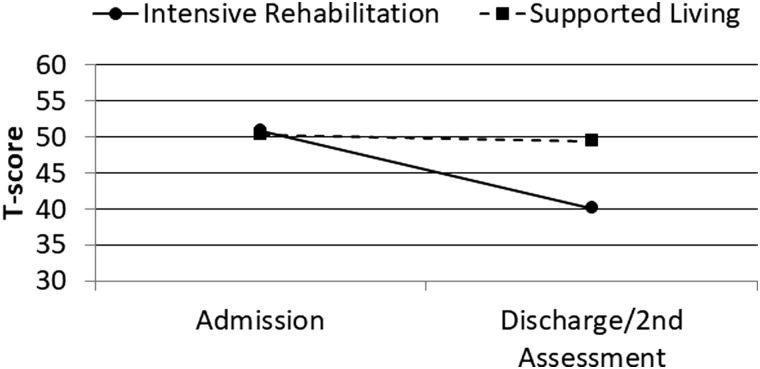
Admission and discharge/second assessment MPAI-4 Total T-scores by program category. MPAI-4, Mayo-Portland Adaptability Inventory-4.

**FIG. 2.**
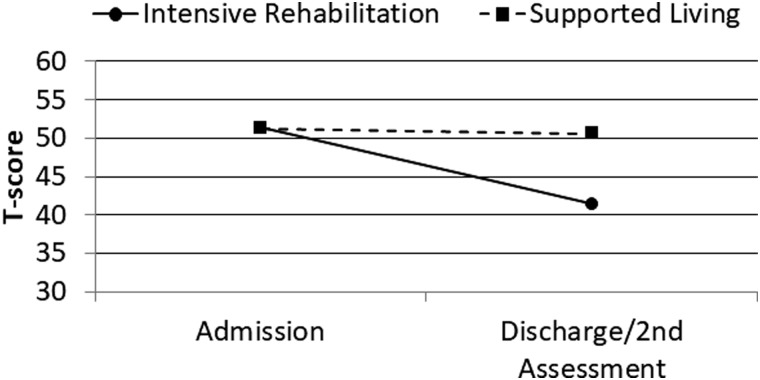
Admission and discharge/second assessment MPAI-4 Ability Index T-scores by program category. MPAI-4, Mayo-Portland Adaptability Inventory-4.

**FIG. 3.**
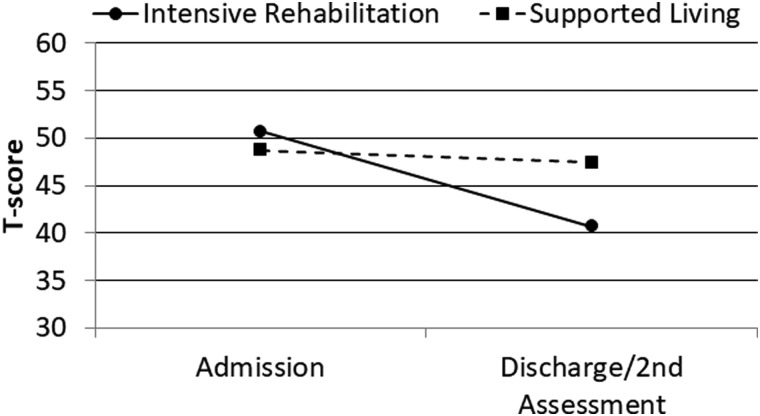
Admission and discharge/second assessment MPAI-4 Adjustment Index T-scores by program category. MPAI-4, Mayo-Portland Adaptability Inventory-4.

**FIG. 4.**
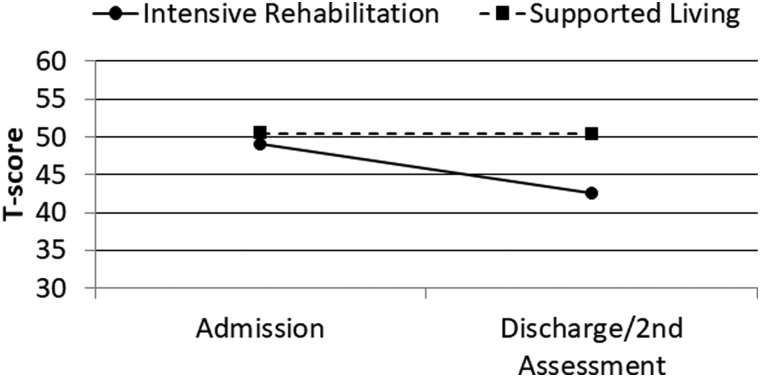
Admission and discharge/second assessment MPAI-4 Participation Index T-scores by program category. MPAI-4, Mayo-Portland Adaptability Inventory-4.

MPAI-4 Index T-scores were subsequently analyzed using similar ANCOVA procedures with similar results. After covarying admission MPAI-4 Index T-score, age at injury, and log chronicity, program category was significantly associated with MPAI-4 discharge/second assessment T-score for the Ability Index (*F* = 49.43; *p* < 0.001; partial eta^2^ = 0.11), Adjustment Index (*F* = 25.20; *p* < 0.001; partial eta^2^ = 0.06), and Participation Index (*F* = 53.76; *p* < 0.001; partial eta^2^ = 0.12). Change over time for these variables is illustrated in Figures 1–4.

Percentages achieving an MCID or better and an RCID or better were significantly greater over the course of program participation for those in the IRR programs than for those in RSL programs (Table 2).

**Table 2.** Percent Achieving MCID and RCID by Program Category

Variable	IRR (*n* = 256)	RSL (*n* = 169)	X^2^
MCID or better	74.6%	17.8%	131.85 (*p* < 0.001)
RCID or better	53.5%	11.8%	75.92 (*p* < 0.001)

MCID, minimal clinically important difference; RCID, robust clinically important difference; IRR, Intensive Residential Rehabilitation; RSL, Residential Supported Living.

### Intensive Residential Rehabilitation program type comparisons

#### Characteristics on admission

Analysis of variance revealed no differences among IRR program types in length of stay (LOS), age at injury, age on admission, or log_10_ chronicity. IRR program types also did not differ in sex, injury type, or funding source. However, on closer examination, there was a larger proportion of Caucasians in the Neurobehavioral program (84.2%) than in the Neurorehabilitation (64.0%) or Day Treatment (68.8%) programs relative to other racial/ethnic groups (*X*^2^ = 8.21; *p* = 0.017). IRR program types also differed on admission MPAI-4 Ability Index (*F* = 8.28; *p* < 0.001), Adjustment Index (*F* = 20.10; *p* < 0.001), Participation Index (*F* = 7.23; *p* = 0.001), and Total Score (*F* = 15.08; *p* < 0.001). Post-hoc least significant difference comparisons among the three program types indicated that greater disability for the Neurobehavioral group was recorded on the Ability Index, Participation Index, and Total Score than for the Neurorehabilitation group, which, in turn, showed greater disability on these measures than the Day Treatment group. The Neurobehavioral group showed greater disability on the Adjustment Index on admission than either of the other two program types. Admission characteristics of each IRR program type are detailed in Table 3.

**Table 3.** Demographic, Injury, and MPAI-4 Variables on Admission by IRR Program Type

Variable	Neurorehabilitation (*n* = 161)	Neurobehavioral (*n* = 57)	Day treatment (*n* = 38)
Sex (% male)	89.4%	91.2%	89.5%
LOS (days)	110.12 (84.73)	132.95 (86.35)	103.84 (88.92)
Mean (SD) age at Injury (years)	42.24 (15.40)	41.86 (16.58)	43.53 (11.63)
Mean (SD) age at admission (years)	44.06 (14.93)	44.07 (14.94)	46.21 (11.85)
Mean (SD) days post-injury at admission	630.96 (1395.29)	816.95 (1478.02)	554.29 (908.22)
Race/ethnicity			
Caucasian	64.0%	84.2%	65.8%
African American	8.7%	3.5%	5.3%
Hispanic	21.1%	10.5%	26.3%
Asian	1.2%	0.0%	0.0%
Native American	2.5%	0.0%	0.0%
Other	2.5%	1.8%	2.6%
Injury type			
Closed	85.0%	87.5%	73.7%
Open	1.9%	1.8%	2.6%
Stroke	8.1%	1.8%	13.2%
Anoxia	3.1%	5.4%	2.6%
Other	1.9%	3.5%	7.9%
Funding source^a^			
Workers' Compensation	57.1%	52.6%	55.3%
Commercial/no-fault	13.0%	10.5%	21.1%
Private pay/lien	19.3%	12.3%	13.2%
Military	9.9%	17.5%	10.4%
Public	.7%	7.1%	0.0%
Mean (SD) MPAI-4 T-score at admission			
Ability Index^a^	51.29 (10.55)	55.23 (11.38)	45.97 (11.43)
Adjustment Index^a^	48.67 (10.84)	59.09 (13.44)	46.50 (11.26)
Participation Index^a^	48.69 (11.01)	52.86 (11.05)	44.42 (8.74)
Total^a^	49.92 (10.91)	57.54 (12.88)	45.16 (11.12)

^a^ Significant difference (*p* < 0.05) between groups.

MPAI-4, Mayo-Portland Adaptability Inventory-4; IRR, Intensive Residential Rehabilitation; LOS, length of stay; SD, standard deviation.

#### Mayo-Portland Adaptability Inventory-4 outcomes

ANCOVAs controlling for admission MPAI-4 T-score, age at injury, and log chronicity showed no significant differences among the IRR program types on discharge MPAI-4 Index and Total T-scores. The ANCOVA on the Ability Index was marginal (*F* = 2.95; *p* < 0.054); however, when correction for multiple comparisons was applied, this difference was no longer significant. Discharge T-scores by program type are displayed in Table 4.

**Table 4.** Discharge MPAI-4 Index and Total T-Score Means (SD) by Program Type and Category

	Ability index	Adjustment index	Participation index	Total score
Neurorehabilitation	41.16 (12.97)	39.63 (13.01)	42.20 (9.59)	39.41 (13.42)
Neurobehavioral	43.26 (11.64)	44.61 (14.55)	45.02 (9.63)	43.75 (13.37)
Day Treatment	39.82 (12.84)	39.13 (15.42)	39.97 (8.34)	37.68 (13.75)
Intensive Rehabilitation	41.43 (12.66)	40.66 (13.90)	42.50 (9.52)	40.12 (13.56)
Supported Living	50.63 (13.38)	47.36 (11.65)	50.37 (10.90)	49.52 (11.71)

MPAI-4, Mayo-Portland Adaptability Inventory-4; SD, standard deviation.

A smaller proportion of Day Treatment program participants (60.5%) achieved an MCID or better at discharge than participants in the Neurorehabilitation (77.0%) or Neurobehavioral (77.2%) programs. However, this difference was not statistically significant (*X*^2^ = 4.67; *p* = 0.097). Similarly, a small proportion of those in Day Treatment (39.5%) achieved an RCID or better than in Neurorehabilitation (54.0%) or Neurobehavioral (61.4%) programs, but this difference was also not statistically significant (*X*^2^ = 4.46; *p* = 0.108). In both cases, the relatively small number of Day Treatment participants (*n* = 38) makes the estimate of the percentage of successful cases less statistically reliable.

### Comparison of matched samples of Intensive Residential Rehabilitation and Residential Supported Living participants

Chronicity differed between participants in IRR and RSL programs and was significantly related to outcome. In order to control for this variable more rigorously than by using it as a covariate, we identified a sample of participants in both program categories matched on chronicity as well as age and sex. This sample consisted of 58 individuals (29 in each program category). There were no significant differences between IRR and RSL participants on sex, injury type, or ethnicity. Funding source did differ between the two groups (*X*^2^ = 12.24; *p* = 0.007). In the RSL group, 75.8% were funded by Workers' Compensation and 3.5% by military, commercial, or no-fault insurance, whereas in the IRR group 37.9% were funded by Workers' Compensation and with 38.0% by military, commercial, or no-fault insurance. *t*-tests revealed no significant difference between groups on age on admission, age at injury, or log chronicity. LOS was longer in the RSL programs (mean = 5080.72 days) than in IRR programs (mean = 167.24 days; *t* = −10.41; *p* < 0.001). Admission T-score on the Adjustment Index was higher for participants in IRR programs than for those in RSL programs (*t* = 2.68; *p* = 0.01). There were no significant differences between groups on admission on the Ability Index, Participation Index, or Total T-score.

ANCOVA, covarying initial T-score, age at injury, and log chronicity, as in previous analyses, revealed significant differences between IRR and RSL participants at discharge for MPAI-4 Ability Index (*F* = 29.75; *p* < 0.001; partial eta^2^ = 0.360), Adjustment Index (*F* = 16.97; *p* < 0.001; partial eta^2^ = 0.243), Participation Index (*F* = 25.45; *p* < 0.001; partial eta^2^ = 0.324), and Total T-score (*F* = 27.70; *p* < 0.001; partial eta^2^ = 0.343). Probably because error variance was more constrained in this matched sample, effect sizes are notably large. Admission and discharge/second assessment MPAI-4 T-scores are displayed in Table 5.

**Table 5.** Admission and Discharge MPAI-4 Index and Total T-Score Means (SD) by Program Category (Matched Subsample)

	Ability index	Adjustment index	Participation index	Total score
Intensive Rehabilitation				
Admission	57.72 (11.20)	56.21 (7.45)	56.41 (10.55)	57.41 (7.24)
Discharge	46.14 (8.80)	45.34 (11.62)	46.76 (9.64)	45.59 (9.12)
Supported Living				
Admission	56.55 (11.70)	49.28 (11.76)	53.52 (9.42)	53.76 (10.23)
Discharge	55.14 (11.82)	48.28 (12.29)	53.03 (10.15)	52.41 (10.69)

MPAI-4, Mayo-Portland Adaptability Inventory-4; SD, standard deviation.

An MCID or better was achieved by a larger proportion of those in IRR programs (79.3%) than in RSL programs (6.9%; *X*^2^ = 31.00; *p* < 0.001). Similarly, an RCID or better was achieved by larger proportion of IRR participants (69.0%) than RSL participants (6.9%; *X*^2^ = 23.28; *p* < 0.001) (see Table 6).

**Table 6.** Percent Achieving MCID and RCID by Program Category (Matched Subsample)

Variable	IRR (n = 29)	RSL (*n* = 29)	X^2^
MCID or better	79.3%	6.9%	31.00 (*p* < 0.001)
RCID or better	69%	6.9%	23.28 (*p* < 0.001)

MCID, minimal clinically important difference; RCID, robust clinically important difference; IRR, Intensive Residential Rehabilitation; RSL, Residential Supported Living.

## Discussion

Results of this study contribute to the current brain injury rehabilitation effectiveness literature in several important ways. First, results demonstrate that participants in the IRR program significantly improve over the course of treatment and that participants in the RSL group preserved functional ability over the course of 1 year, suggesting that although each program is effective in achieving its intended outcome, the IRR treatment group achieved significantly better outcomes than those in the same setting not receiving the intervention. In order to more rigorously isolate treatment effects of IRR, a subsample of participants matched on time post-injury, age, and sex were analyzed. By controlling for time post-injury, the treatment effect of IRR was strengthened, suggesting that chronicity alone does not account for the variance between the two groups.

Past literature has emphasized the role that chronicity is thought to play in the ability of IRR to produce a significant impact on functional improvement after acquired brain injury. It is important to note that in this study, the mean number of days post-injury at admission for the IRR group was 1.8 years, with a range of 14 days to 39.1 years. For the RSL group, the mean time post-injury at admission was 7.6 years with a range of 43 days to 36.1 years. The overlap between the two groups on time post-injury suggests that other factors besides time-post injury determine program entry, including funding and target outcome of the admission. The results of the matched sample comparisons speaks to the fact that IRR improves functional outcomes for individuals regardless of timing post-injury. Overall, these findings suggest that individuals do benefit from the range of services provided and underscores the need to understand the individualized nature of post-hospital treatment planning and timing of that treatment.

The MPAI-4 as an outcome measure for post-acute rehabilitation is widely accepted. However, it is insensitive to more subtle changes, changes in very low functioning individuals, or changes that occur in higher-functioning individuals with very specific goals across a limited number of functions. It does not directly measure objective or subjective burden-of-care experiences.

Information about the longer-term outcomes of these programs cannot be provided, thus limiting the ability to make assumptions about the durability of these program outcomes.

Although participants in each program type received the same type of intervention framework with customized components based on individual need, this study did not examine the number of specific services received by each participant or the duration of intervention. Thus, we are not able to glean any information about potential dose-response impacts on outcomes. Similarly, predictors of outcome for each group are not addressed. Future research endeavors will address these limitations.

One final limitation that is important to note is that although we attempted to more closely approximate an experimental design than past studies, the retrospective analysis clearly lacks the critical features of a true experiment, for example, prospective random assignment to control and experimental conditions.

Chronic disability as a result of acquired brain injury is an expensive burden on society. Rehabilitation that is effective in restoring independence and productivity and reimbursement of such rehabilitation is of critical importance in reducing overall burden on the individual, the family, and society.

Intensive rehabilitation programs result in significant functional improvements whereas supported living programs support stable functioning over time. The effectiveness of IRR is more clearly demonstrated when treatment sample is homogenously residential versus studies that have mixed residential and community-based IRR samples. Improvement in functional ability is significant for those who participate in residential IRR programs, regardless of chronicity.

There is a continued need for collaboration across providers to collectively explore impact and expected outcomes associated with various post-inpatient rehabilitation programs.
